# *In vitro* patterning of pluripotent stem cell-derived intestine recapitulates *in vivo* human development

**DOI:** 10.1242/dev.138453

**Published:** 2017-03-15

**Authors:** Yu-Hwai Tsai, Roy Nattiv, Priya H. Dedhia, Melinda S. Nagy, Alana M. Chin, Matthew Thomson, Ophir D. Klein, Jason R. Spence

**Affiliations:** 1 Department of Internal Medicine, Gastroenterology, University of Michigan Medical School, Ann Arbor, MI 48109, USA; 2 Institute for Human Genetics andDepartment of Pediatrics, University of California, San Francisco, San Francisco, CA 94143, USA; 3 Program in Craniofacial Biology and Department of Orofacial Sciences, University of California, San Francisco, San Francisco, CA 94143, USA; 4 Department of Surgery, University of Michigan Medical School, Ann Arbor, MI 48109, USA; 5 Department of Cell and Developmental Biology, University of Michigan Medical School, Ann Arbor, MI 48109, USA; 6 Center for Systems and Synthetic Biology, University of California, San Francisco, San Francisco, CA 94143, USA; 7 Center for Organogenesis, University of Michigan Medical School, Ann Arbor, MI 48109, USA

**Keywords:** Human, Intestine, Organoid, Patterning, Pluripotent stem cells

## Abstract

The intestine plays a central role in digestion, nutrient absorption and metabolism, with individual regions of the intestine having distinct functional roles. Many examples of region-specific gene expression in the adult intestine are known, but how intestinal regional identity is established during development is a largely unresolved issue. Here, we have identified several genes that are expressed in a region-specific manner in the developing human intestine. Using human embryonic stem cell-derived intestinal organoids, we demonstrate that the duration of exposure to active FGF and WNT signaling controls regional identity. Short-term exposure to FGF4 and CHIR99021 (a GSK3β inhibitor that stabilizes β-catenin) resulted in organoids with gene expression patterns similar to developing human duodenum, whereas longer exposure resulted in organoids similar to ileum. When region-specific organoids were transplanted into immunocompromised mice, duodenum-like organoids and ileum-like organoids retained their regional identity, demonstrating that regional identity of organoids is stable after initial patterning occurs. This work provides insights into the mechanisms that control regional specification of the developing human intestine and provides new tools for basic and translational research.

## Abstract (precis)

**Summary:** Human embryonic stem cell-derived intestinal organoids can be patterned into duodenum-like or ileum-like tissue, recapitulating *in vivo* human development.

## INTRODUCTION

The mature gastrointestinal tract is a highly compartmentalized organ with distinct regions that serve specific roles in digestion, absorption, hormone secretion and immunity. Many studies have detailed the complex reciprocal interactions between the endoderm-derived epithelium and the mesoderm-derived stroma for establishing regional identity along the anterior-posterior axis of the gut tube in the early embryo (Dessimoz et al., 2006; Duluc et al., 1994, 2001; Kedinger et al., 1998; Ratineau et al., 2003; Sherwood et al., 2011; Wells and Melton, 2000). However, very little is known about the mechanisms in place after intestinal specification that pattern the duodenum, jejunum and ileum, which make up the regions of the small intestine from proximal to distal.

One of the earliest requirements for intestinal development is the establishment of embryonic posterior identity (Wells and Melton, 1999; Zorn and Wells, 2007). Although the embryo receives anterior-posterior (A-P) positional information prior to and during gastrulation, posterior endoderm identity is not irreversibly specified until after gastrulation (Ho and Kimmel, 1993; Horb and Slack, 2001). At this stage, there are several signaling pathways that promote the posterior patterning of the vertebrate embryo. The WNT and FGF signaling pathways play a central role in establishing the posterior axis of the vertebrate embryo, and Caudal homeobox (Cdx) genes, which are transcription factors that regulate intestinal specification, development and maintenance of regional identity, are targets of these signaling pathways (Beck and Slack, 1999; Beland et al., 2004; Bradley et al., 2000; Cox and Hemmati-Brivanlou, 1995; Dale et al., 1992; Dessimoz et al., 2006; Domingos et al., 2001; Erter et al., 2001; Gao et al., 2009; Grainger et al., 2010; Greco et al., 1996; Gregorieff et al., 2004; Hollyday et al., 1995; Huelsken et al., 2000; Ikeya and Takada, 2001; Isaacs et al., 1998; Keenan et al., 2006; Kiecker and Niehrs, 2001; Lekven et al., 2001; Lickert et al., 2000; Liu et al., 1999; Marvin et al., 2001; McLin et al., 2007; Northrop and Kimelman, 1994; Parr and McMahon, 1995; Pownall et al., 1996; Satoh et al., 2006; Verzi et al., 2010, 2013).

FGF and/or WNT signaling controls posterior fate and intestinal lineage commitment in mouse or human pluripotent stem cells (PSCs) (Cao et al., 2011, 2015; Hannan et al., 2013; Sherwood et al., 2011; Tamminen et al., 2015), and a combination of WNT and FGF signaling can induce an intestinal fate in human definitive endoderm (DE), which gives rise to human intestinal organoids (McCracken et al., 2011; Spence et al., 2011). Although it is clear that hPSC-derived intestinal organoids are small intestinal in identity (Finkbeiner et al., 2015b; Spence et al., 2011; Watson et al., 2014), their exact regional identity (duodenum, jejunum, ileum) is unclear. Here, we sought to clarify the regional identity of organoids, and to determine whether organoids could be patterned into different regions of the intestinal tract. Given that the posterior endoderm of the developing embryo is exposed to higher concentrations and longer durations of growth factors (Arnold and Robertson, 2009), we hypothesized that the duration of exposure to active FGF and WNT signaling would control regional intestinal identity. In this study, we take advantage of hESC-derived intestinal organoids to test this hypothesis. Our results demonstrate that the duration of exposure to active WNT/β-catenin (using CHIR99021, a GSK3β inhibitor) and FGF (FGF4) signaling results in gene and protein expression profiles that are consistent with tissue that has been patterned into proximal (duodenum) or distal (ileum) small intestine, respectively. To validate our findings, we used both candidate gene expression analysis to compare organoids with the human fetal intestine and an unbiased transcriptome-level approach. To determine whether ‘patterned’ organoids retain their regional identity *in vivo*, we transplanted organoids into mice, which allows maturation of organoids into adult-like tissue (Finkbeiner et al., 2015b; Watson et al., 2014).

Taken together, our findings shed light on the mechanisms that control regional identity of the developing human intestine. Regionally specified organoids provide a platform for uncovering the genes and signaling pathways that are responsible for common congenital malformations or for functional adaptation of the adult gastrointestinal tract following injury. We suggest that the impact of regional identity is an important consideration in such studies.

## RESULTS

### Regional identity markers in human fetal intestine

**Fig. 1.**
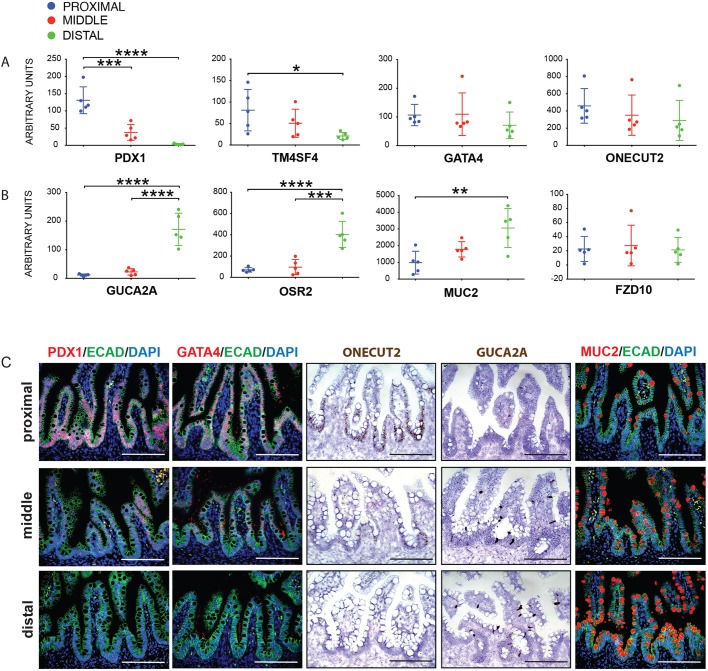
**Identification of regionally expressed molecular markers in the human fetal intestine.** (A) Genes known to be enriched in the proximal developing mouse intestine, including *PDX1*, *GATA4*, *TM4SF4* and *ONECUT2* were examined in different regions of the human fetal intestine (*n*=5 individual biological specimens; proximal, blue; middle, red; distal, green). (B) Genes know to be enriched in the distal developing mouse intestine, including *GUCA2A*, *OSR2*, *MUC2* and *FZD10* were examined in different regions of the human fetal intestine (*n*=5 individual biological specimens; proximal, blue; middle, red; distal, green). (C) Enrichment of PDX1 and GATA4 protein as assessed by immunofluorescence and of *ONECUT2* mRNA as assessed by *in situ* hybridization was confirmed in the proximal region of the fetal intestine, whereas *GUCA2A* mRNA and MUC2 protein were enriched in the distal fetal intestine when assessed by *in situ* hybridization and immunofluorescence, respectively. Scale bars: 200 μm.

Many genes with differential regional identity in the small intestine have been identified in the adult human and mouse intestine, where gene expression reflects adult region-specific intestinal function, as well as in the developing murine intestine. However, in the developing/fetal human intestine, genes corresponding to adult function are expressed at very low levels (Finkbeiner et al., 2015b), and therefore adult-stage markers do not faithfully identify regional identity in the embryo. Fortunately, several regional identity markers have been described in the fetal mouse intestine (Battle et al., 2008; Dusing et al., 2001; Gao and Kaestner, 2010; Sherwood et al., 2009). To identify a cohort of markers that are regionally expressed in the human intestine, we assessed expression of genes and proteins orthologous to those enriched in embryonic mouse regions by qRT-PCR, *in situ* hybridization and immunofluorescent staining (Fig. 1; *n*=5, independent biological samples ranging from 14-19 weeks of gestation). Intestines from human fetuses were obtained and divided into thirds, corresponding to the proximal, middle and distal regions of the small intestine. We observed that *PDX1* and *TM4SF4* were enriched in the proximal intestine, similar to the embryonic mouse proximal intestine (Sherwood et al., 2011), whereas the expression of *GATA4* and *ONECUT2* showed non-statistically significant trends of higher expression in the proximal human intestine (Fig. 1A). Of note, some individual samples had more pronounced region-specific expression of *GATA4* and *ONECUT2* when technical replicates were examined (Fig. S1), and regional-specific expression was confirmed for *ONECUT2* using *in situ* hybridization (Fig. 1C), suggesting that region-specific gene expression may be dynamic over time, or may vary significantly between biological specimens; however, additional studies at each time point will be required to more conclusively assess biological variation or time-dependent changes. *Guca2a*, *Osr2*, *Muc2*, *Fzd10*, *Cib2* and several Hox genes have higher distal gene expression levels in mice (Gao and Kaestner, 2010; Sherwood et al., 2009). In the human fetal intestine, *GUCA2A*, *OSR2* and *MUC2* showed increased expression in the distal small intestine (Fig. 1B), along with *HOXB6* (Fig. S1). We confirmed proximal enrichment of PDX1 and *ONECUT2* and distal enrichment of MUC2 and *GUCA2A* using immunofluorescence and *in situ* hybridization (Fig. 1C). Together, these data identify a cohort of molecular markers that are regionally expressed in the human fetal small intestine and demonstrate that some of these regional identifiers are conserved between the mouse and human fetal small intestine.

### Prolonged WNT/FGF signaling distalizes hESC-derived intestinal organoids

**Fig. 2.**
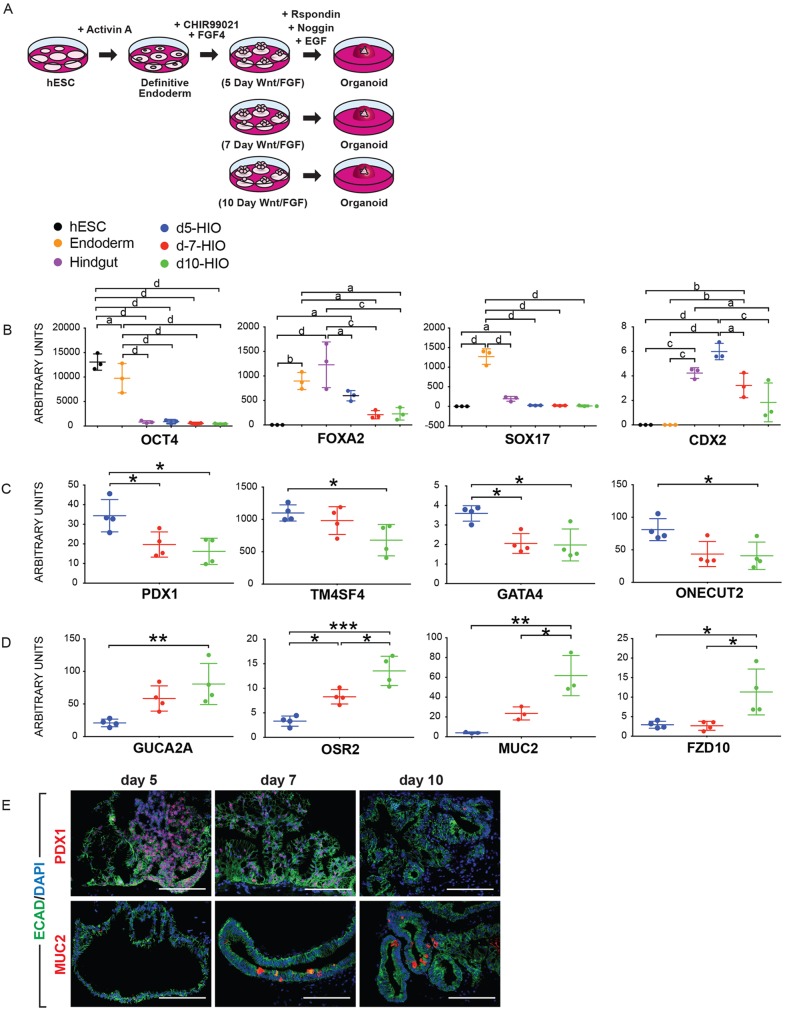
**Human intestinal organoids are patterned by FGF and WNT signaling.** (A) Schematic of experimental design showing spheroids generated in culture over increasing periods of time. (B) Expression of *OCT4*, *FOXA2*, *SOX17* and *CDX2* during differentiation in undifferentiated hESCs, in endoderm and hindgut (4 days after FGF4/CHIR99021), and in organoids derived from d5, d7 and d10 cultures (d5, blue; d7, red; d10, green). (C) Markers shown to be enriched in the human fetal duodenum (Fig. 1), including *PDX1*, *GATA4*, *TM4SF4* and *ONECUT2* were examined in d5, d7 and d10 organoids. (D) Markers shown to be enriched in the human fetal ileum (Fig. 1), including *MUC2*, *OSR2*, *MUC2* and *FZD10* were examined in d5, d7 and d10 organoids. (E) Immunofluorescence demonstrated that PDX1 protein expression was enriched in d5 organoids, whereas MUC2 protein expression was enriched in d7 and d10 organoids. Scale bars: 200 μm.

To examine the effects of WNT and FGF signaling on developing human intestinal tissue, we took advantage of the hESC-derived intestinal organoid culture system (Spence et al., 2011). hESCs were exposed to activin A for 3 days to induce endoderm, which was then exposed to CHIR99021/FGF4-enriched media to induce mid/hindgut spheroid formation, as previously described (Finkbeiner et al., 2015a,b; Xue et al., 2013). Spheroids began to bud from monolayer cultures after 4 days and continuously generated new spheroids for over 10 days; however, incubation beyond 10 days resulted in far fewer spheroids (data not shown). Spheroids were collected from the cultures after 5 days (d5), 7 days (d7) or 10 days (d10), and embedded into matrigel (Fig. 2A). Spheroids were expanded into larger human intestinal organoids for 30-35 days in intestinal growth medium containing EGF, noggin and R-spondin 2 (Fig. 2A). qRT-PCR performed on tissues collected at progressive stages of organoid differentiation, including undifferentiated hESCs, definitive endoderm, hindgut tissue after 4 days of CHIR99021/FGF4 and organoids generated after d5, d7 and d10, showed the expected stage-specific mRNA expression of pluripotency genes (*OCT4*), endoderm genes (*FOXA2*, *SOX17*) and the mid-hindgut and intestinal specification gene *CDX2* (Fig. 2B).

We next evaluated the effects of exposing human DE to CHIR99021 and FGF4 for different lengths of time, and examined the expression of region-specific markers of the developing human intestine identified in Fig. 1. Similar to the human fetal intestine, we found that d5 human organoids had significantly higher expression of the proximal identity marker genes *PDX1*, *TM4SF4*, *GATA4* and *ONECUT2* compared with d7 and d10 organoids (Fig. 2C). Conversely, distally enriched intestinal genes *GUCA2A*,* OSR2* and *MUC2* were expressed at significantly higher levels in d10 organoids compared with d5 and d7 organoids (Fig. 2D). Interestingly, whereas *FZD10* was not regionally expressed in the distal human fetal intestine, it was significantly higher in d10 organoids (Fig. 2D). Although further exploration of this observation is warranted, one possible explanation for this discrepancy is that organoids may represent an earlier stage of development than the human fetal intestine used in this study. Immunostaining confirmed that PDX1 was more abundant in d5 organoids, and that MUC2 was more abundant in d7 and d10 organoids (Fig. 2E).

To further validate our findings, we conducted a series of additional experiments (Fig. S2). We treated endoderm for 5 days with FGF4 plus CHIR99021 (500 ng/ml FGF4, 2 μM CHIR99021), and then varied the concentration of CHIR99021 for the next 5 days, or alternatively, removed CHIR99021 and added IWP2, a WNT inhibitor, or the FGF and ERK inhibitors SU5402 or U0126, respectively (Fig. S2). Spheroids from all conditions were embedded in matrigel and expanded into organoids for 30 days, and were then compared for regional gene expression. As expected, d5 and d10 organoids demonstrated region-specific gene expression when grown in standard conditions (as in Fig. 2, 500 ng/ml FGF4+2μM CHIR99021). However, when CHIR99021 concentrations were reduced, d10 organoids expressed much higher levels of proximal markers but this did not lead to reduced posterior marker gene expression. Interestingly, when WNT signaling was blocked between d5 and d10 (FGF4+IWP2), expression of proximal genes was enhanced and distal gene expression was reduced when compared with d5 and d10 organoids, respectively. These data suggest that, during distalization, one of the roles of WNT/β-catenin may be to repress proximal genes while inducing posterior genes (Fig. S2). In total, these experiments show that prolonged exposure to high levels of CHIR99021 is required for expression of distal intestinal markers.

### Regional identity is maintained over time *in vitro*

It is unclear whether organoids change over time in culture, and it is also unknown whether regional identity might also be determined by the length of time spent in culture. To test this, we generated d5, d7 and d10 organoids, and examined them after 1 month and after 90 days in culture (compare Fig. 2 with Fig. S3). Although some individual regional identity markers changed over time in culture, the trends were similar at the two different time points: d5 organoids had more abundant proximal marker expression with low distal marker expression whereas d10 organoids had low proximal marker expression with enriched distal marker expression.

### Whole-transcriptome profiling by RNA sequencing demonstrates that organoids are patterned into proximal and distal intestine

Because the expression patterns of several markers of human fetal proximal-distal intestinal identity suggested that d5 organoids are similar to human duodenum and d10 organoids are similar to human fetal ileum (Figs 1 and 2), we next took an unbiased approach to confirm these results. We conducted RNA-sequencing (RNAseq) at different stages of differentiation, including undifferentiated hESCs (H9 hESC line), DE and organoids grown for 30 days from each stage of spheroid formation [5 day organoids (OD5), 7 day organoids (OD7) and 10 day organoids (OD10)].

**Fig. 3.**
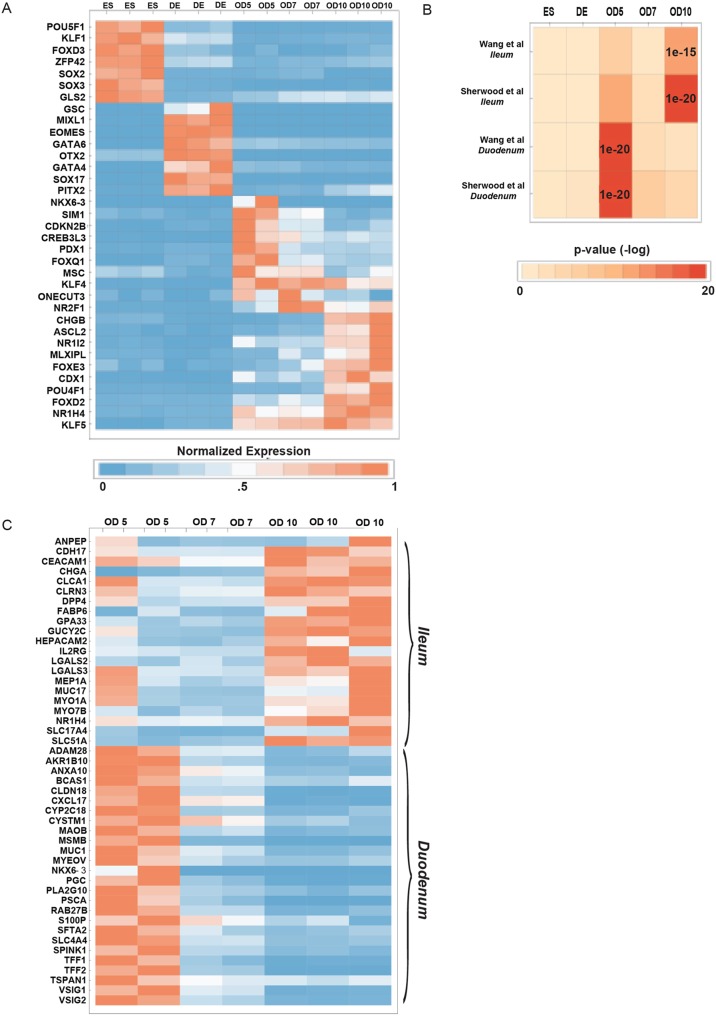
**Bioinformatic identification of stage-enriched genes and comparison with published datasets.** (A) Non-negative matrix factorization was used to identify stage-enriched genes. A normalized and curated heatmap shows representative genes (the full list is in Table S1). ES, human embryonic stem cells; DE, definitive endoderm; OD5, day 5 organoids; OD7, day 7 organoids; OD10, day 10 organoids. (B) Enriched genes in d5, d7 and d10 organoids were compared with published lists of genes whose expression is regionally restricted to the duodenum or the ileum. A hypergeometric test was used to determine the level of significance of overlapping gene sets. (C) Heatmap of representative genes found to overlap in patterned organoids and in published datasets shows enrichment for ileal genes in d10 organoids and for duodenal genes in d5 organoids.

As a first step towards an unbiased assessment of the hypothesis that d5, d7 and d10 organoids are patterned into proximal or distal intestine, we determined unique stage-specific gene expression patterns in each of our RNAseq datasets. Using non-negative matrix factorization (NNMF) (Brunet et al., 2004), we identified gene expression programs that were highly enriched at one stage among the various conditions (Fig. 3A, Table S1). Each program corresponds to a cohort of genes that are statistically enriched at only one stage. In order to determine whether d5 gene expression programs corresponded to duodenal genes and d7/10 gene expression programs corresponded to ileal genes, we conducted a hypergeometric test to compare organoid gene expression programs (enriched gene sets) against regional identity gene sets of the fetal mouse and human intestine previously identified by microarray (Sherwood et al., 2009; Wang et al., 2015) (Fig. 3B). In this analysis, it was important to compare gene expression programs with data obtained from fetal, as opposed to adult, intestine because, as we have recently demonstrated, organoids resemble fetal intestine and do not express many of the genes found in the adult organ (Finkbeiner et al., 2015b). This analysis revealed that d5 gene sets had statistically significant overlap with genes expressed in the duodenum of the mouse (Sherwood et al., 2009; ‘Sherwood duo’) and human (Wang et al., 2015; ‘Wang duo’) (*P*<1.0×10^−18^ for both), whereas the d10 gene set had statistically significant overlap with genes expressed in the ileum of the mouse (Sherwood et al., 2009; ‘Sherwood ileum’; *P*<1.0×10^−18^) and human (Wang et al., 2015; ‘Wang ileum’; *P*<2.22×10^−16^) (Fig. 3B, Table S2). Importantly, we could not resolve whether d7 organoids are similar to jejunum based on Sherwood et al. and Wang et al., because the gene sets exclusively expressed in this region were very small and did not allow us to perform the statistical comparisons with confidence. Thus, we have limited our conclusions to d5 and d10 organoids. Overlapping genes identified in the duodenum/d5 organoid and ileum/d10 organoids comparisons (Fig. 3B) were further plotted as a heatmap (Fig. 3C). As a control, we also compared d5 and d10 gene expression programs against genes that are enriched in the human fetal colon (Wang et al., 2015). Here, we found no statistically significant overlap, adding confidence to our conclusion that d5 and d10 organoids are most similar to human duodenum and ileum, respectively (Fig. S4).

### Confirming regional identity markers in human fetal tissue

**Fig. 4.**
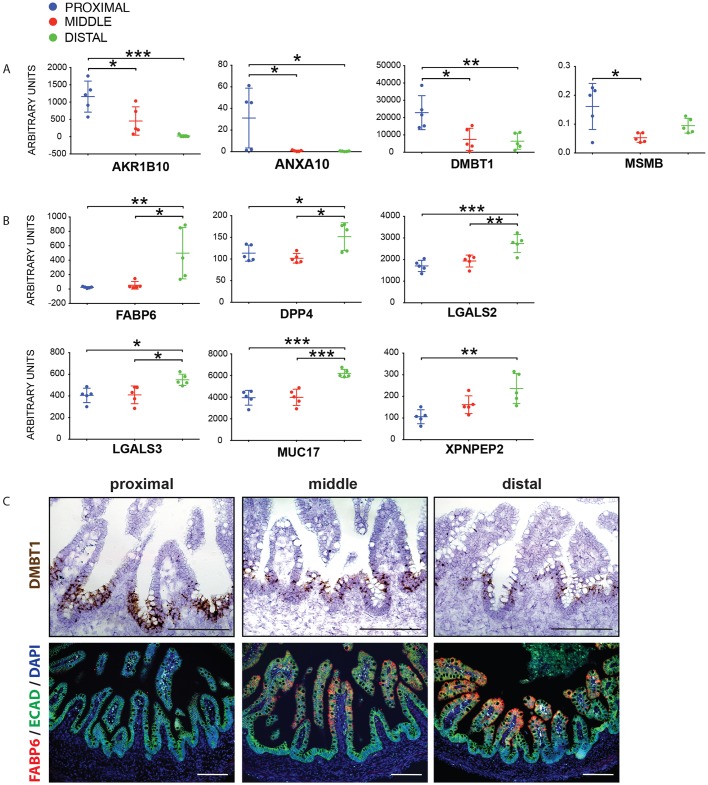
**Validation of region-enriched genes in human fetal tissue.** (A) qRT-PCR showing genes enriched in the human fetal proximal small intestine (*n*=5 individual biological specimens; proximal, blue; middle, red; distal, green). (B) qRT-PCR showing genes enriched in the human fetal distal small intestine. (C) *In situ* hybridization of *DMBT1* showing stronger expression in the proximal small intestine, and immunohistochemistry of FABP6 showing more abundant protein staining in the middle/distal regions of the human small intestine. Scale bars: 200 μm.

The analyses presented thus far demonstrate that increased culture time is well correlated with progressive distalization of intestinal organoids. However, we also needed to grapple with several caveats at this point, including: (1) organoids grown *in vitro* lack many cell types found in the endogenous intestine (e.g. vasculature, enteric neurons, immune cells); (2) genes regionally expressed in the human intestine were identified in tissue grown in culture that supported growth of the epithelium only and that was enriched for stem/progenitor cells but had few differentiated cell types (Wang et al., 2015); and (3) several of the regionally expressed genes were identified in mouse as opposed to human (Sherwood et al., 2009). Therefore, we set out to further validate our findings by investigating expression of genes identified in Fig. 3 (Table S2) in full thickness human fetal intestine. In total, our results had identified 33 ‘duodenal’ genes and 26 ‘ileal’ genes that were consistent across datasets. Of note, several of the genes identified in our unbiased analysis were identified in our candidate approach (Fig. 1), including *PDX1*, *TM4SF4*,* ONECUT2* and *GUCA2A*. We further confirmed that four additional duodenal genes were enriched in the proximal human fetal intestine compared with the distal, and six ileal genes were enriched in the distal compared with the proximal human fetal intestine (Fig. 4, Fig. S5). Although other genes did not show enrichment across the different regions of the human fetal intestine (Figs S6 and S7), it is important to note that examination of technical replicates between intestine regions of individual samples often showed region-specific gene expression (compare Fig. S6 with Fig. S7). Thus, it is possible that our approach has identified additional region-specific markers but that our analysis was not sufficiently powered at individual gestational stages to draw strong conclusions. In addition, it is interesting that many of the genes enriched in the human fetal intestine in a regional manner were regulated in a graded, as opposed to a binary ‘on/off’, manner, and only a few genes appeared to be expressed exclusively in one region or another.

### Regional identity is maintained in patterned organoids transplanted *in vivo*

**Fig. 5.**
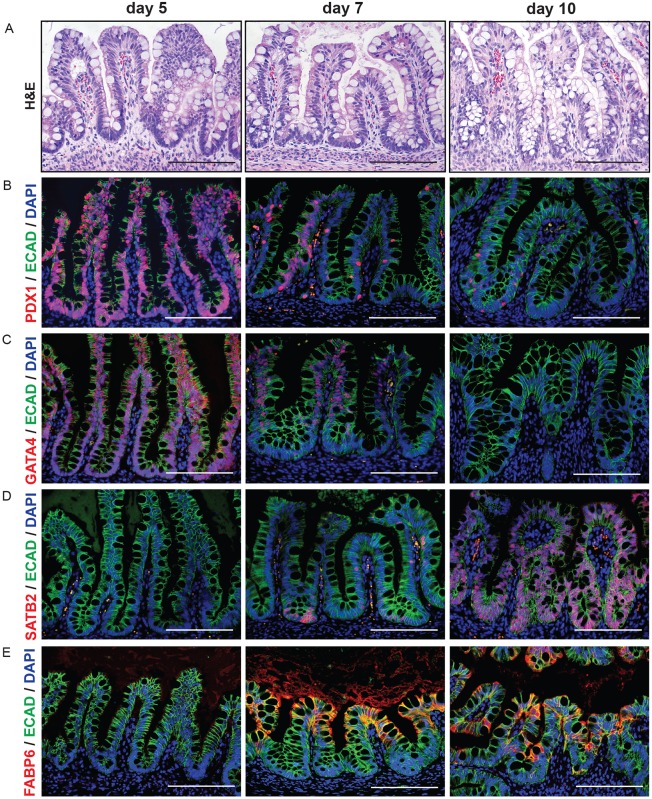
**Organoids retain regionalization after maturation *in vivo.*** (A) d5, d7, d10 organoids were harvested after maturation *in vivo*. Hematoxylin and Eosin staining reveals that transplanted tissue possesses villus- and crypt-like domains. (B) PDX1 is most highly enriched in d5 organoids. (C) GATA4 is most highly enriched in d5 organoids. (D) SATB2 is most highly enriched in d10 organoids. (E) FABP6 is enriched in both d7 and d10 organoids. Scale bars: 200 μm.

Organoids grown *in vitro* are similar to the fetal intestine, and upon transplantation into immunocompromised mice, they become more mature at the cellular, molecular and functional levels (Finkbeiner et al., 2015b; Watson et al., 2014). Transplantation also causes morphological remodeling such that the simple epithelium of the organoid *in vitro* gains crypt-villus structure similar to that of the native adult human intestine (Finkbeiner et al., 2015b; Watson et al., 2014). Thus, in order to confirm that the patterned organoids maintained their regional identity when transplanted *in vivo*, we generated d5, d7 and d10 organoids, and transplanted them under the kidney capsule of immunocompromised NSG mice (*n*=5 per group; Fig. S8). Organoids were allowed to engraft and mature for 10 weeks, and were then harvested for histological and immunohistochemical analysis (Fig. 5, Figs S9 and S10). Low-magnification Hematoxylin and Eosin staining revealed crypt-villus architecture in d5, d7 and d10 transplanted organoids (Fig. 5A, Fig. S9A). As expected, proliferation, marked by Ki67, was restricted to the crypt domains in all transplanted tissues (Fig. S9). In addition, CDX2 and the brush border enzymes sucrose isomaltase (SI) and dipeptidyl peptidase IV (DPPIV), which are expressed throughout the small intestine, were similarly expressed in all regionalized organoids (Fig. S10). By contrast, many proteins known to be expressed in the proximal small intestine (duodenum) were restricted to the d5 organoids, with very little or no expression in d10 organoids (Middendorp et al., 2014; Uhlén et al., 2010), including PDX1, GATA4, FABP2 and LCT (Fig. 5B,C, Fig. S9C). Similarly, proteins previously shown to be enriched in the ileum were more abundant in the d10 organoids, including the transcription factor SATB2, which is expressed at high levels in the adult colon and at lower levels in the distal ileum and FABP6 (Fig. 5D,E) (Uhlén et al., 2010; Wang et al., 2015). In addition, goblet cells, marked by MUC2, were much more abundant in d7 and d10 organoids (Fig. S9E,F).

## DISCUSSION

In this study, we provide evidence that the duration of exposure to FGF4 and CHIR99021 is able to specify the regional identity of organoids from human PSCs *in vitro.* This work sheds new light onto the regional identify of intestinal organoids grown using published methods (Finkbeiner et al., 2015b; McCracken et al., 2011; Spence et al., 2011; Watson et al., 2014), as it has been unclear whether these most closely represented a specific region of the intestine. Here, we show that organoids used in published work most closely represent duodenum, and we demonstrate that prolonged activation of FGF and WNT signaling patterns the intestine into increasingly distal tissue. The notion that duration of exposure to signaling pathway activation can influence identity came from our understanding that, as the embryo develops, the most caudal region of the embryo is exposed to higher levels of growth factor signaling relative to more proximal regions, and that this signaling is prolonged as high concentrations of ligands are maintained distally as the embryonic axis lengthens (Arnold and Robertson, 2009). However, whether or not intestinal patterning was dependent on growth factor signaling activity and time of exposure was unclear. Our findings provide evidence that variables, including concentration and length of exposure, are important for establishing intestinal regional identity.

Furthermore, the current studies also address several practical issues that arise from working with *in vitro*-derived tissues. One important issue that our work raises relates to understanding the strengths and limitations of different organoid model systems in order to ensure that they are employed in the correct context (Chen et al., 2014; Finkbeiner et al., 2015b, 2012; Leslie et al., 2014; Rodansky et al., 2015; Watson et al., 2014; Xue et al., 2013). For example, a number of reports have demonstrated that hPSC-derived organoids are small intestinal in nature and contain Paneth cells (Finkbeiner et al., 2015a,b; Spence et al., 2011; Watson et al., 2014), which are not present in the colon (Rothenberg et al., 2012). However, the regional identity of organoids generated using the same or similar methods to ours has been confused in other research, including recent reports stating that organoids are tissues ‘resembling human proximal colon’ (Engevik et al., 2015a,b). The current work underscores the observation that organoids, as originally described (Spence et al., 2011), are most similar to duodenum.

In addition to clarifying the nature of these *in vitro*-derived models, another important implication of the current work is the ability to begin generating different regions of the intestine for specific purposes. For example, a dangerous condition in human neonates called necrotizing enterocolitis (NEC) most often affects the ileum. When using organoids to model diseases such as NEC *in vitro*, or for generating tissue engineered small intestine for therapeutic purposes, it will be important to generate organoids that best match the appropriate region of the intestine (Finkbeiner et al., 2015a; Howell and Wells, 2011; Levin et al., 2013; Ullrich et al., 2012).

Given the inability to functionally explore developmental processes in human fetal tissue, using organoids as a surrogate model for human intestine development provides a path for better understanding of the developmental cues responsible for human fetal intestinal regionalization. Previously, little to nothing was known about this process in the human; this study provides the first evidence that the combined signaling activity of FGF and WNT plays a role in establishing regional intestinal identity. In the future, *in vitro*-derived regionalized small intestinal organoids can be used to study specific gene expressions programs and to better understand genes that play important roles in the development of congenital intestinal disease. From a regenerative medicine perspective, regionalized organoids will provide more reliable building blocks for replacement purposes.

## MATERIALS AND METHODS

### hESC lines, human tissue and mice

#### hESCs

All work with hESCs was reviewed and approved by the University of Michigan human pluripotent stem cell research oversight committee (HPSCRO). The hESC cell line H9 (WA09, NIH stem registry #0062) was obtained from the WiCell Research Institute. Karyotypically normal cell lines were used for all experiments.

#### Human tissue

Normal, de-identified human intestinal tissue was obtained from the University of Washington Laboratory of Developmental Biology, and was approved by the University of Michigan institutional review board.

#### Animal use

All mouse work was reviewed and approved by the University of Michigan Committee on Use and Care of Animals (UCUCA).

### Differentiation of hESCs

Differentiation of hESCs and organoids was carried out as previously published, with minor modifications (Finkbeiner et al., 2015b; Leslie et al., 2014; McCracken et al., 2011). In brief, endoderm was generated by adding activin A (100 ng/ml) for 3 consecutive days in Roswell Park Memorial Institute 1640 (RPMI-1640) media supplemented with 0%, 0.2% and 2.0% HyClone FBS. FGF4 (500 ng/ml) plus CHIR99021 (2 μM) were then added to endoderm cultures and medium was replaced daily for 10 days. In our original protocol, spheroids were collected on d4 (McCracken et al., 2011; Spence et al., 2011), whereas in the current work, spheroids were collected on d5, d7 and d10, embedded in matrigel and overlaid with growth medium, as previously described (Finkbeiner et al., 2015b), containing RSPO2-conditioned medium (Bell et al., 2008), noggin-conditioned medium (Heijmans et al., 2013) and EGF. All experiments were conducted on organoids expanded for 30-35 days *in vitro*.

### qRT-PCR

Briefly, RNA isolation was performed using MagMAX^TM^-96 Total RNA Isolation Kit (Ambion, AM1830). A SuperScript VILO cDNA synthesis kit (ThermoFisher, 11754250) was used to make cDNA from 200 ng RNA. cDNA levels were detected using QuantiTect SYBR Green (Qiagen, 608056). Relative gene expression was plotted as Arbitrary Units, using the following formula: [2^(housekeeping gene Ct-gene Ct)]×10,000. All primers sequences are listed in Table S3.

### Histology, immunofluorescence and *in situ* hybridization

Immunofluorescence was carried out as previously described (Dye et al., 2015; Rockich et al., 2013) using antibodies outlined in Table S4. *In situ* hybridization was performed using the RNAscope 2.0 HD detection kit, and with commercially available mRNA probes outlined in Table S4, according to the standard protocol provided. All incubations were performed at 40°C in a HybEZ hybridization system oven (Advanced Cell Diagnostics, 310010). All immunostaining or *in situ* hybridization was conducted on at least three independent biological specimens (three independent human fetal samples or three independent organoids), and immunostaining images shown in the figures are representative images unless otherwise noted in the text.

### Statistical analysis

For statistical analysis, data are expressed as the median of each sample set. Each data point in the plots represents an independent biological sample. For organoid experiments, each independent biological sample is comprised of three to five organoids pooled together. All organoid experiments were conducted on at least three independent biological replicates, and each experiment was repeated on at least two separate occasions (independent experiments). For human fetal tissue, all analysis was conducted on five independent biological replicates. One-way ANOVA was used for statistical analysis, except for Fig. S2, which used an unpaired *t*-test. Analyses were carried out with GraphPad Prism 5.0 software. Each data point is presented, with the middle line representing the mean, the error bars representing±s.e.m. In all figures, *P<0.05, **P<0.01, ***P<0.001, ****P<0.0001, except for Fig. 2B where a=*P*<0.05, b=*P*<0.01, c=*P*<0.001, d=*P*<0.0001. For experiments in the supplemental data where individual human fetal samples are plotted (Figs S1, S5, S7), the intestine (*n*=1 biological sample per time point) was divided into proximal, middle and distal regions, and each region was measured and cut into 1 cm lengths. RNA was isolated from three to five different segments from each of the proximal, middle and distal regions (*n*=3-5 technical replicates).

### RNA sequencing

RNA was isolated directly from tissue culture plates using the MagMAX^TM^-96 Total RNA Isolation Kit according to the manufacturer's protocol. RNA concentration and purity were assessed using Nanodrop spectrophotometer and bioanalyzed using Agilent RNA 6000 Nano Kit (260/280>1.7 and 260/230>1.7, RIN>8). Samples were stored at −80°C. cDNA libraries were generated using TrueSeq Kit (Illumina). Sequencing was then performed on HiSeq 2000 (Illumina, 100 bp, single-end reads). In all, 42 samples were run using two flow cells and four samples per lane. RNA sequencing data are available in the ArrayExpress database (www.ebi.ac.uk/arrayexpress) under accession number E-MTAB-4168.

### RNA sequencing and data analysis

Raw mRNA-seq reads were aligned to hg19 exome constructed from UCSC gff files. Reads were aligned using Bowtie2 v 2.1.0 (Langmead and Salzberg, 2012) with the following options -D 25-R 3-N 1-L 20-i S 1 0.50 local. Transcript counts were normalized to fragments per kilobase of transcript per million mapped reads (FPKM) by applying UCSC transcript lengths and the number of collected reads per sample. Transcripts with a maximum FPKM of less than one across all samples were discarded.

Gene expression programs were assembled using non-negative matrix factorization (Lee and Seung, 1999). Prior to NNMF, the gene expression values were normalized by the mean gene expression value of each gene across all samples. This step removes expression bias and focuses analysis on gene expression variance during the process of differentiation. Following normalization, NNMF was performed both on the entire genome and on DNA-binding proteins as derived through PFAM annotations. DNA-binding proteins provide a reduced set of interpretable genes for comparison with existing literature. NNMF was performed with 10 replicates, and the highest scoring factorization was selected as the global dictionary of programs.

To assess enrichment of literature gene sets in NNMF programs, gene sets were assembled from Wang et al. (2015) and Sherwood et al. (2009), and gene set enrichment was performed as described previously (Mootha et al., 2003; Subramanian et al., 2005) to determine enrichment in NNMF programs. A gene was determined to be a member of an NNMF-derived program if the gene loading was two standard deviations above the mean loading in the NNMF program. Statistical significance for enrichments was calculated using the hyper-geometric test. Principal component analysis was performed using standard procedures on the FPKM and mean normalized gene expression data.

### Kidney capsule transplantation

Kidney capsule transplantation was carried out as previously described (Finkbeiner et al., 2015b; Watson et al., 2014). All organoids were transplanted after being grown *in vitro* for 30-35 days. For each group (d5, d7, d10), organoids were transplanted into five different mice; each transplanted mouse was considered an independent biological specimen (*n*=5 per group) (Fig. S7). Transplanted organoids were harvested after 10 weeks.
